# Network heterogeneity and seizure generation

**DOI:** 10.1186/1471-2202-16-S1-P302

**Published:** 2015-12-18

**Authors:** Sima Mofakham, Christian G Fink, Victoria Booth, Michal R Zochowski

**Affiliations:** 1 Biophysics program, University of Michigan, Ann Arbor, MI , USA; 2 Department of Physics & Astronomy and Neuroscience Program, Ohio Wesleyan University, Delaware, OH, USA; 3 Departments of Mathematics and Anesthesiology, University of Michigan, Ann Arbor, MI, USA; 4 Department of Physics, University of Michigan, Ann Arbor, MI, USA; 5 The R.B. Zajonc Institute for Social Studies, Stawki , Warsaw, Poland

## 

It has been shown that seizures occur more frequently at the transition from wake to sleep, or from one stage of sleep to another. Acetylcholine (ACh) is a neuromodulator that controls wake and sleep stages, and is present at high levels during waking and is absent in slow wave sleep (SWS). ACh has also been shown to switch the excitability as measured with Phase response curves (PRC) of pyramidal cells from Type 2 to Type 1. In general, Type 1 neurons are integrating type with high-excitability while Type 2 have lower excitability but higher capacity for synchronization. We investigate the effect of non-uniform cholinergic modulation, such as might occur at sleep/wake transitions, on the propensity for neuronal synchronization in large-scale networks of Hodgkin*-*Huxley models for cortical pyramidal cells. The interplay between the cellular properties and network connectivity in a heterogeneous network of Type 1 and Type 2 neurons can strongly affect network spatio-temporal dynamics. The focus of this research is to detect conditions that promote synchrony and seizure like activity in a mixed network of Type 1 and Type 2 neurons. Here we investigate inhomogeneous networks built of neurons that have non-identical connectivity properties. Namely every cell has an individual ratio of local and long distance synaptic connections. We show that even if the structure of the network is identical (i.e. identical adjacency matrix) there is a differential network-wide synchronization propensity depending on which neurons have Type II cellular properties.

